# pH-sensitive supramolecular self-assembled peptide hydrogel for the treatment of esophageal cancer

**DOI:** 10.3389/fphar.2024.1453422

**Published:** 2024-10-24

**Authors:** Gaobing Ye, Shiyao Luo, Hajra Zafar, Honglei Ge, Binbin Liu, Nan Wang, Yu Jin, Miao Wang, Xu Chen, Xiaoming Ye

**Affiliations:** ^1^ Affiliated Yueqing Hospital, Wenzhou Medical University, Wenzhou, Zhejiang, China; ^2^ State Key Laboratory of Natural Medicines, School of Pharmacy, China Pharmaceutical University, Nanjing, Jiangsu, China; ^3^ School of Pharmacy, Shanghai Jiao Tong University, Shanghai, China

**Keywords:** esophageal cancer, pH-responsive, peptide, hydrogel, doxorubicin

## Abstract

Esophageal cancer is one of the most common cancers in the world, ranking sixth in cancer-related mortality. Doxorubicin (DOX), as a classic broad-spectrum, non-specific small-molecular anti-tumor drug, has achieved widespread use, including in the treatment of esophageal cancer. However, due to its strong cardiotoxicity, poor tumor-targeting ability, and short half-life, the clinical application of DOX has been greatly limited. In this research, we designed and successfully synthesized a peptide sequence IEIIIK (IEK for short) with excellent pH responsiveness. Under physiological conditions (pH 7.4), the peptide can encapsulate DOX and self-assemble into a stable hydrogel (DOX-IEK) through hydrophobic and electrostatic interactions. After being injected into the acidic tumor microenvironment, the protonation degree of alkaline amino acid lysine increased and the negative charge of glutamate decreased, directly leading to enhanced electrostatic repulsion and subsequent hydrogel dissociation. Released DOX can accumulate in tumor tissue and achieve anti-tumor efficacy. More importantly, the hydrogel can act as a drug reservoir for sustained drug release, improving the drug targeting ability, prolonging the duration of drug administration to compensate for the short half-life of DOX, and reducing systemic toxicity. Ideal anti-tumor efficacy has been achieved in both the *in vitro* and *in vivo* experiments.

## 1 Introduction

Esophageal cancer is currently one of the most common cancers in the world, leading to 509,000 deaths each year and ranking sixth in cancer-related mortality (Bray et al., 2018). According to the World Health Organization, approximately half of the cases occur in China. The main histological type is squamous cell carcinoma, and the overall 5-year survival rate of patients is less than 20% (Kumari et al., 2015).

Doxorubicin (DOX), as a broad-spectrum, non-specific small-molecule chemical anticancer drug of anthracycline, has achieved widespread curative effects on a variety of cancers such as esophageal cancer, breast cancer, osteosarcoma, and leukemia with basically satisfactory treatment efficacy. However, due to strong cardiotoxicity, poor tumor-targeting ability, short half-life, and tumor resistance, the clinical application of DOX has been greatly limited (Rivankar, 2014). At present, a variety of auxiliary nanocarriers have been developed for the drug delivery of DOX, such as liposomes, micelles, and nanoparticles, but these carriers also have certain limitations, such as poor drug-loading capacity, toxic metabolites, or a tendency to trigger immune responses (Rostamabadi et al., 2019; Duan et al., 2020; Yang et al., 2016). Therefore, in order to solve the abovementioned problems, it is urgent to develop a sustained and controlled-release drug delivery system with high drug-loading capacity and biocompatibility.

Hydrogel is a type of material with a three-dimensional network structure (Cai et al., 2020). In the early stage of the development of hydrogel, the 3D networks were usually formed by covalently crosslinked polymers, including polyethylene glycol (Cha et al., 2011), polyvinyl alcohol (Kim et al., 2015), polyacrylamide (Ye et al., 2024), and chitosan (Zhang et al., 2023; Lv et al., 2024). Although the costs of these chemically crosslinked hydrogels were extremely low, due to the presence of trace amounts of harmful reagents or catalysts, certain potential safety hazards still existed, limiting their application in biology (Urakami and Guan, 2008). Among various types of hydrogels, polypeptide hydrogel is a hydrogel with a three-dimensional fiber network structure formed by crosslinking of peptides through physical or chemical bonds with advantages such as high water content, rich micropore structure, adjustable mechanical stability, excellent biocompatibility, excellent injectability, and tissue-like elasticity. In addition, polypeptide hydrogels can be easily modified through side-chain and main-chain modification to achieve specific applications. These characteristics endow peptide hydrogels with extensive applications in the field of biomedicine (Du et al., 2015; Katyal et al., 2020; Sathaye et al., 2015), such as drug delivery (Zhu et al., 2022; Feng et al., 2023), tissue engineering (Xue et al., 2023), and wound healing (Hao et al., 2023). Among them, supramolecular self-assembled peptide hydrogels can be formed by non-covalent interactions between polypeptides, such as hydrogen bonding, π-π stacking, hydrophobic interaction, and electrostatic interaction (Dou et al., 2017; Li et al., 2024). The preparation process does not require additional crosslinking reagents, making the peptide hydrogels easy to degrade and enhancing their responsiveness to environmental stimuli such as ions, pH, light, solvents, and enzymes (Dou et al., 2017; Song et al., 2023).

**SCHEME 1:**
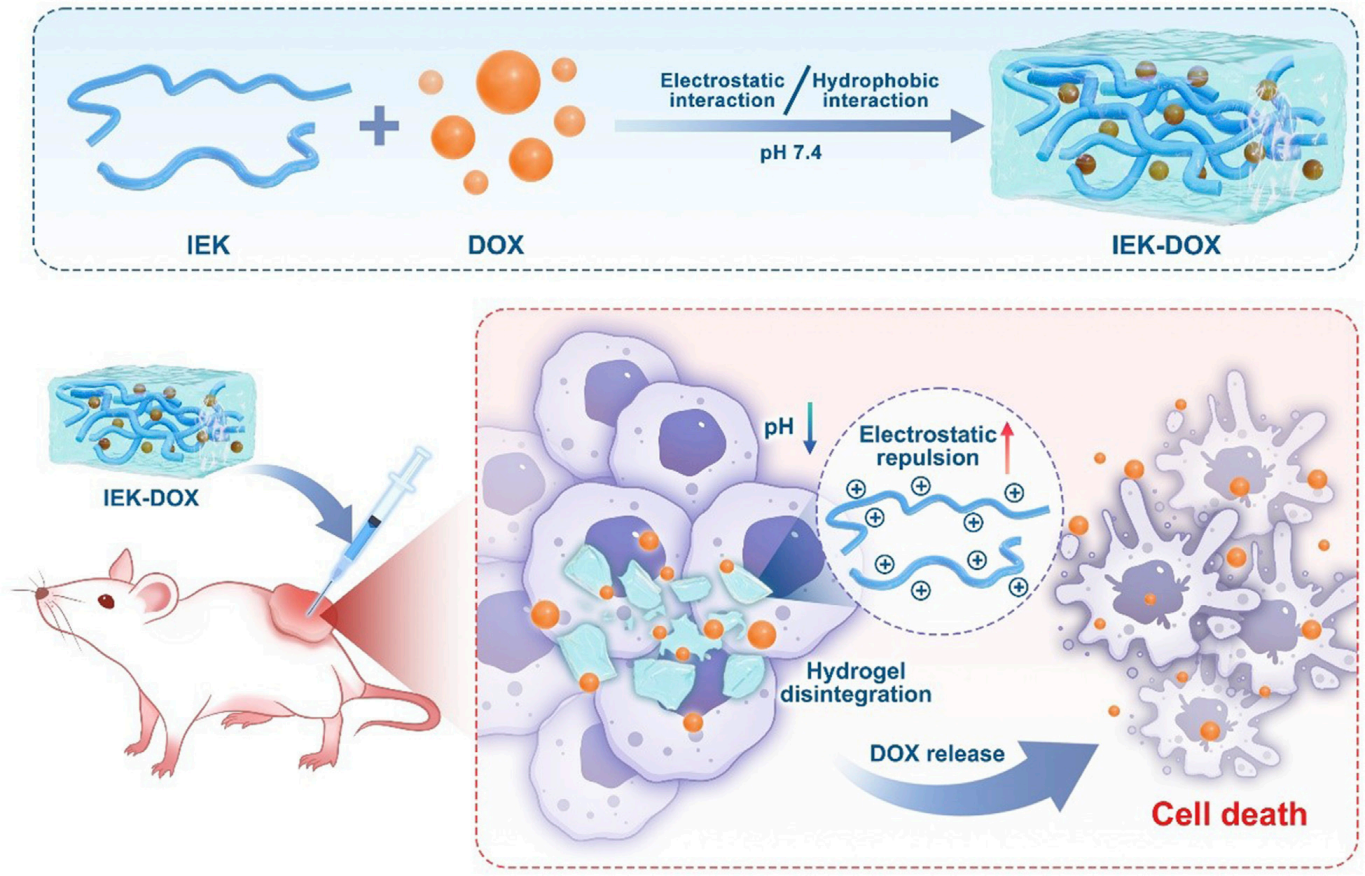
Schematic diagram of the pH-responsive DOX-loaded IEK peptide hydrogel for the efficient treatment of esophageal cancer.

Based on the abovementioned information, we developed a pH-responsive IEIIIK peptide (Scheme 1). The peptide can encapsulate DOX and self-assemble into a stable hydrogel under physiological conditions through a hydrophobic interaction induced by isoleucine and electrostatic interactions between glutamate and lysine. After being injected into the acidic tumor microenvironment, the protonation degree of alkaline amino acid lysine increased and the negative charge of glutamate decreased, directly leading to enhanced electrostatic repulsion and subsequent hydrogel dissociation. Released DOX can accumulate in tumor tissue and achieve anti-tumor efficacy. More importantly, the hydrogel can act as a drug reservoir for sustained drug release, improving drug-targeting ability, prolonging the duration of drug administration to compensate for the short half-life of DOX, and reducing systematic toxicity.

## 2 Materials and methods

### 2.1 Materials

Fmoc-Rink resin, Fmoc-protected amino acids, and 1-hydroxybenzotriazole (HOBT) were obtained from GL (Shanghai, China). EDTA–pancreatin at a concentration of 0.25% was purchased from Beyotime Biotechnology (Shanghai, China), DMEM and 1,640 medium were purchased from Gibco (United States), and doxorubicin hydrochloride (DOX) was purchased from Aladdin (Shanghai, China).

AKR esophageal cancer cell line and HEEC human normal esophageal epithelial cell line were all purchased from Huatuo Biotechnology Co., Ltd (Shenzhen, China).

### 2.2 Ethical approval statement

NOD/SCID mice were purchased from Jiangsu Aniphe BioLab to verify the *in-vivo* anti-tumor efficacy of our drug delivery system. Mice were placed in IVC cages and fed every day. Surrounding temperature and relative humidity were 25°C and 40%–60%, respectively. When injected with tumor suspension or a drug, mice were anesthetized by urethane to minimize suffering. When euthanasia was performed, mice were placed in a closed container and euthanized by injecting CO_2_ with a final concentration of 30% into the container. All animal experiments were carried out in accordance with the guidelines approved by the Laboratory Animal Ethics Committee of Affiliated Yueqing Hospital of Wenzhou Medical University (Ethics approval number: YQYY202400127), and the authors have adhered to the ARRIVE guidelines.

### 2.3 Synthesis of the IEK peptide

The IEK peptide was synthesized by solid-phase peptide synthesis (Supplementary Figure S1). The synthesized peptide was purified by reversed-phase high-performance liquid chromatography (RP-HPLC). Liquid chromatography–mass spectrometry technology (LC-MS) was utilized to analyze the molecular weight of the purified peptide.

### 2.4 Preparation and properties of the IEK peptide hydrogel

#### 2.4.1 Preparation of the IEK hydrogel

The IEK peptide, at a concentration of 10 mg/mL, was dissolved in deionized water. NaOH (1 M) was utilized to adjust the pH of the solution to 7.4. The process of the IEK solution forming a stable, transparent hydrogel was observed.

#### 2.4.2 TEM and DLS

The blank IEK hydrogel was diluted 10 times with deionized water under acid and neutral conditions, and the suspension was then dropped onto a copper mesh. The filter paper was used to absorb the excess sample, dried, and stained with phosphotungstic acid to observe the microstructure of peptide hydrogel using a transmission electron microscope (TEM). The dynamic light scattering (DLS) method was used to measure the fiber length of the peptide hydrogel.

#### 2.4.3 Circular dichroism analysis

Circular dichroism (CD) spectroscopy (JASCO, Oklahoma, United States) was used to analyze the influence of pH on the secondary structure of the blank IEK hydrogel. The CD parameters were as follows: 1 s response time, 50 nm/min scanning speed, and 269–190-nm wavelength range.

#### 2.4.4 Rheological property

In this experiment, a rotating rheometer was used to test the rheological characteristics of the blank IEK hydrogel. A dynamic strain sweep (1 Hz fixed frequency and 0.1%–100% strain sweep range) was performed to determine the linear range of hydrogel’s viscoelasticity. Next, a dynamic frequency sweep was performed with a 0.1% strain and a 0.1–100 rad/s frequency sweep range. Finally, a cyclic strain sweep was performed. The frequency was fixed at 1 Hz. The strain was repeatedly switched as 0.1%–50%–0.1%–50%–0.1%; the sweep time after each transformation was 120 s. By repeatedly applying high and low strains to the IEK peptide, the self-healing ability and injectability of the IEK hydrogel were investigated, recording the changes in the storage modulus (G′) and dissipation modulus (G″) of each test.

### 2.5 Drug-loading capacity

Doxorubicin was selected as the model drug. DOX solutions (1 mg/mL) prepared in purified water and IEK powder were directly mixed to obtain homogenous DOX-IEK solutions. The pH was then adjusted to 7.4 by adding 0.2 M NaOH and left to stand for half an hour, observing the change in each tube of the peptide solution from solution to the formation of a stable gel state. The UV/vis spectrum (Supplementary Figure S4) and standard curve of DOX (Supplementary Figure S5) were drawn. The encapsulation rate of Dox is calculated according to the following formula:

$$\text{Encapsulation rate }\left(\%\right)=\frac{\left(total\;amount\;of\;DOX-remaining\;amount\;of\;DOX\right)}{total\;amount\;of\;DOX}\times 100\%.$$

### 2.6 Drug release study of the self-assembled peptide hydrogel

A self-assembled DOX-IEK peptide hydrogel was prepared according to the method described in Section 2.4 and placed in a 1.5-mL EP tube. PBS solutions with different pH values (pH 5.5, 6.5, and 7.4) were added as a release medium. The release medium was removed at different time points (0–144 h), and a fresh release medium was added. The concentration of DOX was measured using an ultraviolet-visible spectrophotometer, and the cumulative release curve of the drug was drawn.

### 2.7 *In vitro* biocompatibility and cytotoxicity

#### 2.7.1 Biocompatibility

HEEC suspension with a density of 5 × 10^4^ cells/mL was prepared. The cell suspension was inoculated into a 96-well plate. The IEK peptide was dissolved in DMEM and incubated with HEEC for 72 h. The CCK-8 Kit was used to evaluate relative cell viability. A volume of 10 μL CCK-8 solution was added and incubated for an additional 1 h, and the absorbance OD value was detected at 450 nm. Relative cell viability was calculated according to the following formula:

$$\text{Cell viability}\left(\%\right)=\frac{\text{OD}\;\text{target}‐\text{OD}\;\text{blank}}{\text{OD}\;\text{control}‐\text{OD}\;\text{blank}}\times 100\%.$$

#### 2.7.2 Cell uptake

AKR cells were incubated with 1,640 (control), DOX-IEK at pH 5.8, and DOX-IEK at pH 7.4 for 4 h (total concentration of DOX was 10 μg/mL). The cells were fixed with 4% polyformaldehyde for 30 min and stained with 5 μg/mL DAPI for 5 min. Fluorescence images were captured using an inverted fluorescence microscope.

#### 2.7.3 *In vitro* antitumor efficacy

Three groups were established: free DOX group, DOX-IEK at pH 5.8, and DOX-IEK at pH 7.4. DOX-IEK hydrogel and 1,640 medium at pH 7.4/pH 5.8 were used as the release medium, which was collected after 48 h of release behavior at 37°C. AKR cells were seeded in a 96-well plate at a density of 5 × 10^4^ cells/mL (100 μL per well) and cultured at 37°C for 24 h. Five concentration gradients were set up, and a measure of 100 μL of the sample solution diluted with medium was added to each well. The final concentrations of DOX in the free DOX group were 0.1, 1, 10, 20, and 50 μg/mL. After 48 h of incubation at 37°C, the CCK-8 reagent was added, and incubation continued for another 1 h. The cell survival rate and IC_50_ value were calculated for each group.

#### 2.7.4 Cell morphology observation

AKR cells were incubated with 1,640 (control), DOX-IEK at pH 5.8, and DOX-IEK at pH 7.4 for 48 h (total concentration of DOX was 10 μg/mL). After incubation, morphology of AKR cells was recorded using an inverted microscope.

### 2.8 *In vivo* anti-tumor efficacy

#### 2.8.1 Biodistribution

Taking DiR as a simulated drug, it was encapsulated in a hydrogel and its distribution *in vivo* was explored using the *in vivo* imaging system. Tumor-bearing mice with a tumor volume of approximately 250 mm^3^ were randomly divided into two groups (three mice in each group and six mice in total): the DiR group and the DiR-IEK group. The injection volumes of free DiR and DiR-IEK hydrogel were all 100 μL/20 g. The mice were anesthetized by i. p injection of 20% urethane solution (dose: 1 g/kg) at 1, 4, 12, 24, 72, and 144 h after administration, and the distribution of DiR in the body was observed. After 144 h, the tumor tissues and main organs (the heart, liver, spleen, lung, and kidney) were collected, and the distribution of DiR was observed.

#### 2.8.2 *In vivo* anti-tumor efficacy

Six-week-old female NOD/SCID mice were selected and inoculated with AKR esophageal cancer cells *in situ* to evaluate the anti-tumor efficacy of the DOX-IEK hydrogel *in vivo*. When the tumor volume reached 100 mm^3^, mice were randomly divided into three groups (5 mice in each group and 15 mice in total) and weighed. Drug was administered by intertumoral injection, and the dose was 100 μL/20 g (mice in the control group were injected with saline). Body weight and tumor volume were measured every day. After 7 days, the mice were placed in a closed container and euthanized by injecting CO_2_ with a final concentration of 30% into the container. Tumor tissues and main organs (the heart, liver, spleen, lung, and kidney) were dissected, weighed, and fixed in 4% paraformaldehyde. H&E staining and TUNEL staining were performed.

The calculation formula of the mouse tumor volume inhibition rate was as follows: V = LW^2^/2 and inhibition rate $\left(\%\right)=\left(1-V_{\text{sample}}/V_{\text{control}}\right)\;*\;100\%$ . L and W were the maximum and minimum tumor diameters, respectively; V_sample_ was the tumor volume in the sample group (Free DOX or DOX-IEK). V_control_ was the tumor volume of the mice in the control group.

### 2.9 Biocompatibility

Healthy mice were divided into two groups (three mice in each group and six mice in total). Mice in the control group were injected with saline, and those in the IEK group were injected with the blank IEK hydrogel. The injection volume was 100 μL/20 g. After 7 days, mice were euthanized (same method as described in Section 2.7.2), and main organs were collected for H&E staining.

### 2.10 Statistical analysis

One-way ANOVA was used to statistically analyze the significance difference between target groups.

## 3 Result and discussion

### 3.1 Preparation of the IEK peptide and IEK hydrogel

**FIGURE 1:**
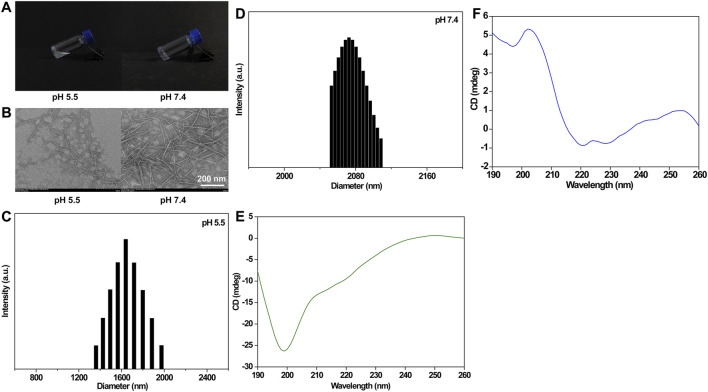
Morphology **(A)** and TEM images **(B)** of the blank IEK peptide hydrogel at pH 5.5 and 7.4. Scale bar: 200 nm. DLS spectrum of the blank IEK peptide hydrogel at pH 5.5 **(C)** and 7.4 **(D)**. Circular dichroism spectrum of the blank IEK peptide hydrogel at pH 5.5 **(E)** and pH 7.4 **(F)**.

The IEIIIK peptide (IEK) prepared by solid-phase synthesis was composed of hydrophobic amino acid isoleucine (I), glutamate (E) (pI = 3.22, negatively charged at pH 7.4), and lysine (K) (pI = 9.74 positively charged at pH 7.4). HPLC showed that the IEK peptide sample had a high purity of 99.5% (Supplementary Figure S2), meeting our experimental requirements. The molecular weight of IEIIIK was 727.96, so the theoretical value of its [M + H]H^+^ was 728.98. LC-MS revealed that the actual value was 729.01, which was extremely close to the theoretical value, proving the successful synthesis of the IEK peptide (Supplementary Figure S3). Subsequently, we focused on exploring its gelation property. IEIIIK dissolved in deionized water formed a transparent acid solution with a concentration of 10 mg/mL. After adjusting the pH to 7.4, strong gelation occurs immediately due to hydrophobic interactions between isoleucine and electrostatic interactions between glutamate and lysine (Figure 1A).

### 3.2 Characterization of the IEK hydrogel

TEM images of the blank IEK peptide hydrogel are shown in Figure 1B. Under acidic conditions, spherical vesicles and nanorods coexist in the hydrogel formed by the IEK peptide. The formed fibers were also relatively short. When under neutral conditions, nanorods could form uniform long fibers, the fibers were entangled and interwoven to form a fiber network, thereby forming a stable hydrogel. DLS showed that peptide molecules aggregated and self-assembled to form slender nanofibers with an average particle size of 2000–2,100 nm at pH 7.4 (Figure 1D). However, when the pH value of the solution decreased to 5.5 (Figure 1C), the distribution of particle size mainly concentrated within the range of 400–700 nm. The abovementioned results indicated that peptide molecules in the neutral microenvironment were attracted by amino acid residues with opposite charges on their molecular chains, resulting in enhanced electrostatic interactions. Peptide molecules aggregate to form slender nanofibers, leading to an increase in particle size distribution. After reducing the pH of the solution, due to the protonation of alkaline amino acids in the peptide molecules, electrostatic repulsion in the system sharply increased, leading to the depolymerization of nanofibers into short segments, resulting in a decrease in the overall particle size. This was consistent with the aforementioned TEM and gelation behavior. The secondary structure of the IEK peptide was further validated by circular dichroism. As shown in Figures 1D, E, the circular dichroism spectrum showed chromatographic peaks in a random coil state at pH 5.5. However, when the pH increased to 7.4, the circular dichroism spectrum illustrated a strong positive peak at 190∼220 nm and a negative peak at 220 nm, indicating that during the process of pH elevation, short peptides gradually changed from random coils to α-helical structures, forming a stable supramolecular hydrogel with a nanofiber network structure.

### 3.3 DOX encapsulation and drug release behavior

**FIGURE 2:**
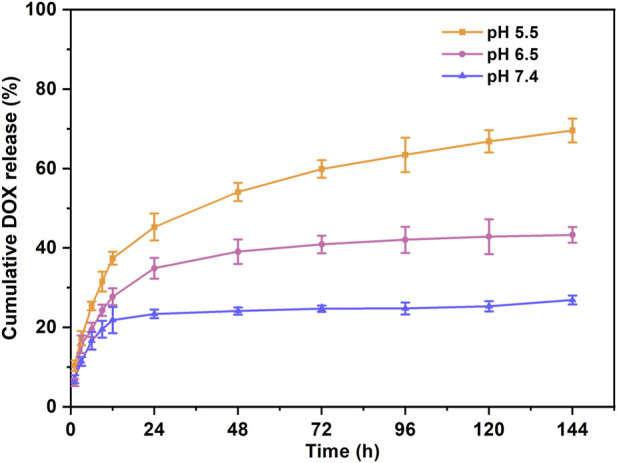
Cumulative release rate of DOX under different pH conditions within 144 h.

When the encapsulated concentration of DOX increased from 1 mg/mL to 3 mg/mL, a stable hydrogel formed in a short time, with a drug encapsulation rate higher than 96%. The release behavior of the DOX-IEK hydrogel displayed significant pH dependence. In the release medium with a pH of 5.8, the cumulative release rate of DOX reached up to 70% within 144 h, while in the medium with a pH of 7.4, it was only 11.12% (Figure 2). This result showed that the dense fiber network structure of the peptide hydrogel slowly disintegrated in the acidic tumor microenvironment, accelerating the release of DOX embedded in the pores of the three-dimensional network structure of the hydrogel.

### 3.4 Rheological property

**FIGURE 3:**
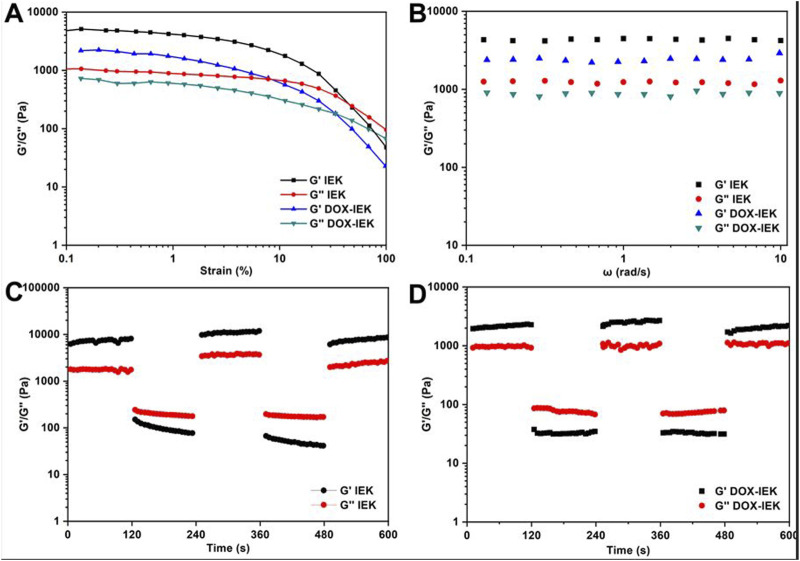
**(A)** Dynamic strain sweep of the blank IEK hydrogel and DOX-IEK. **(B)** Dynamic frequency sweep of the blank IEK hydrogel and DOX-IEK. Circle sweep of the blank IEK **(C)** hydrogel and DOX-IEK **(D)**.

Confirming the solid stability and self-healing property of the IEK peptide hydrogel was essential for the intramural hydrogel injection. As shown in Figure 3A, the elasticity of the hydrogel was represented by the storage modulus G′, and the viscosity was represented by the energy dissipation modulus G″. When G′ was greater than G″, the hydrogel exhibited a solid state without fluidity. On the contrary, when G″ was greater than G, it appeared as a fluid in a liquid state (Li et al., 2022). Dynamic strain scanning results showed that the critical strain value of both blank hydrogel and DOX-loaded DOX-IEK hydrogel was approximately 34%. When the applied strain exceeded the critical strain, G′ started to be smaller than G″, indicating that the hydrogel transformed into a liquid state. In dynamic frequency scanning (Figure 3B), G′ was always much greater than G″, further indicating the formation of a stable hydrogel. Dynamic time scanning showed that G′ was greater than G″ when a low strain (0.1%) was first exerted. When the strain immediately enhanced to 50%, G″ became larger than G′, which was consistent with the previous results. When a high strain (50%) switched to a low strain, G′ and G″ immediately restored to the state close to the initial value (Figures 3C, D). Based on the statistics, we inferred that the IEK hydrogel can be used as an injectable material to deliver anti-cancer drugs, forming a stable drug reservoir at the tumor site to improve the therapeutic effect of DOX.

### 3.5 *In vitro* biocompatibility

**FIGURE 4:**
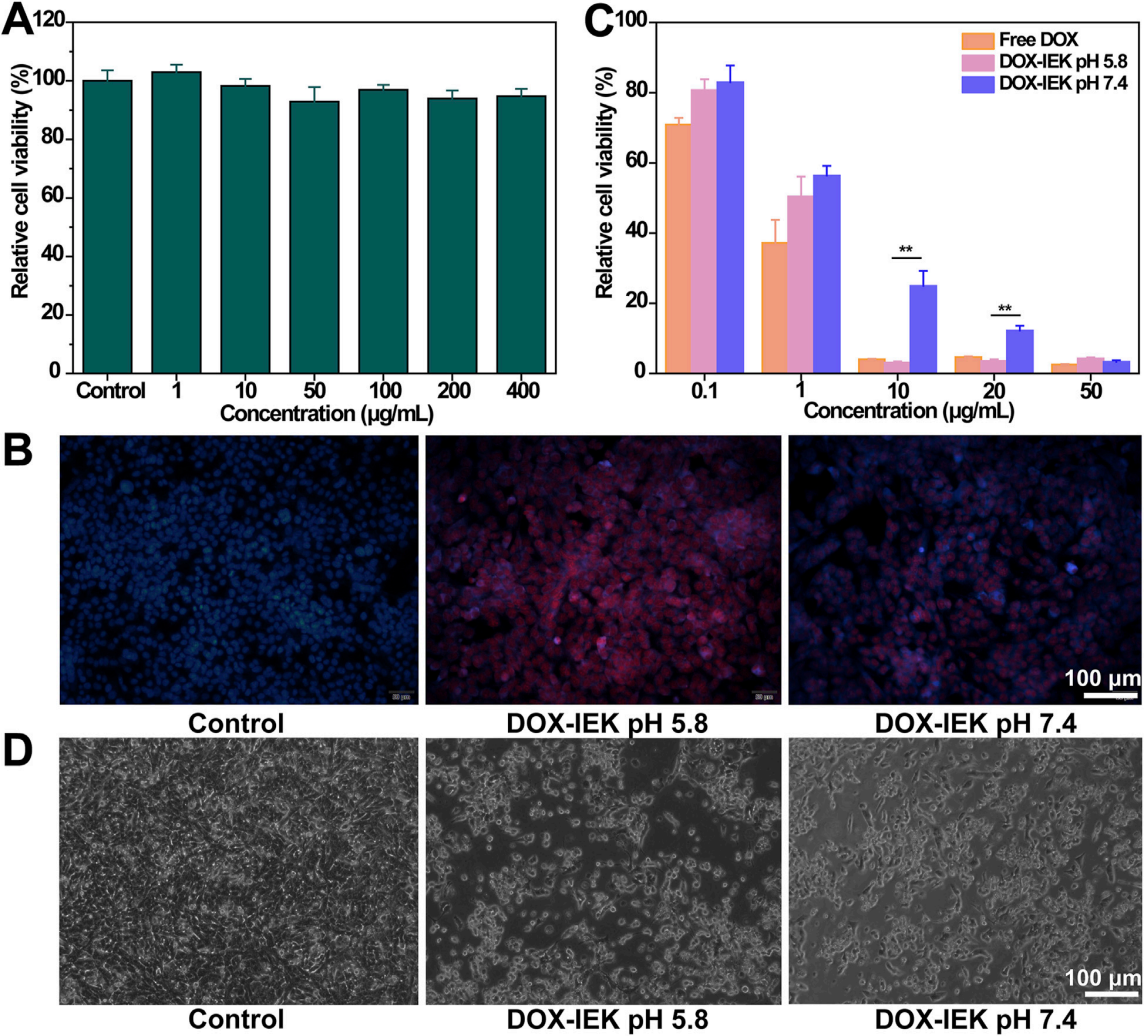
**(A)** Relative cell viability of HEEC cells incubated with the IEK peptide within the range of 0–400 μg/mL for 48 h. **(B)** Fluorescence images of AKR cells after 4 h of incubation with 1640 medium, DOX-IEK at pH 5.8, and DOX-IEK at pH 7.4 for 48 h. Relative cell viability **(C)** and morphology **(D)** of AKR cells co-incubated with 1640 medium, DOX-IEK at pH 5.8, and DOX-IEK at pH 7.4 for 48 h. **, *p* < 0.01.

The CCK-8 assay was performed to detect the biocompatibility of the IEK peptide. The results showed that the survival rate of HEEC cells incubated with the IEK peptide for 72 h was greater than 95% (Figure 4A). The relative cell viabilities of HEEC cells treated with the IEK peptide at concentrations of 1 μg/mL, 10 μg/mL, 50 μg/mL, 100 μg/mL, 200 μg/mL, and 400 μg/mL were 98.23%, 102.30%, 96.10%, 98.94%, 99.91%, and 96.90%, respectively, illustrating that the IEK peptide showed no cytotoxicity and demonstrated excellent cell biocompatibility, making it a promising potential drug delivery carrier for the treatment of esophageal cancer.

### 3.6 *In vitro* anti-tumor efficacy

First, we examined the cell uptake behavior of DOX with different administration. The control group showed no red fluorescence signal (Figure 4B). DOX-IEK at pH 5.8 showed the strongest fluorescence signal, which was much stronger than that of DOX-IEK at pH 7.4 as the acidic microenvironment led to more drug release due to the pH responsiveness of the IEK hydrogel, thus greatly increasing the cellular uptake of DOX.

Subsequently, we examined cytotoxicity to verify anti-tumor efficacy *in vitro*. The inhibition rates of AKR cells treated with different concentrations of free DOX and DOX-IEK under different pH conditions are shown in Figure 4C. As the concentration of doxorubicin increased, the inhibition rate of AKR remarkably increased. Free DOX and DOX-IEK at pH 5.8/7.4 demonstrated that the cytotoxicity toward AKR cells was concentration-dependent. With the increase in DOX concentration, the cytotoxicity of DOX-IEK at pH 5.8 was significantly higher than that of DOX-IEK at pH 7.4 due to more released DOX, which was consistent with the cell uptake experiment. IC_50_ results also showed that DOX-IEK at pH 5.8 had better anti-tumor efficacy (Table 1). Finally, we observed the cell morphology of AKR. As shown in Figure 4D, AKR cells showed a round shape incubated with DOX-IEK at pH 5.8, indicating widespread cell death. However, cell death of AKR cells incubated with the release solution under DOX-IEK at pH 5.8 was relatively less, further illustrating that the medicinal IEK hydrogel had ideal pH responsiveness and *in vitro* anti-tumor efficacy.

**TABLE 1:** IC_50_ values of different drug administrations.

Group	IC_50_ (μg/mL)
Free DOX	0.358
DOX-IEK at pH 5.8	0.612
DOX-IEK at pH 7.4	1.21

### 3.7 *In vivo* biodistribution

**FIGURE 5:**
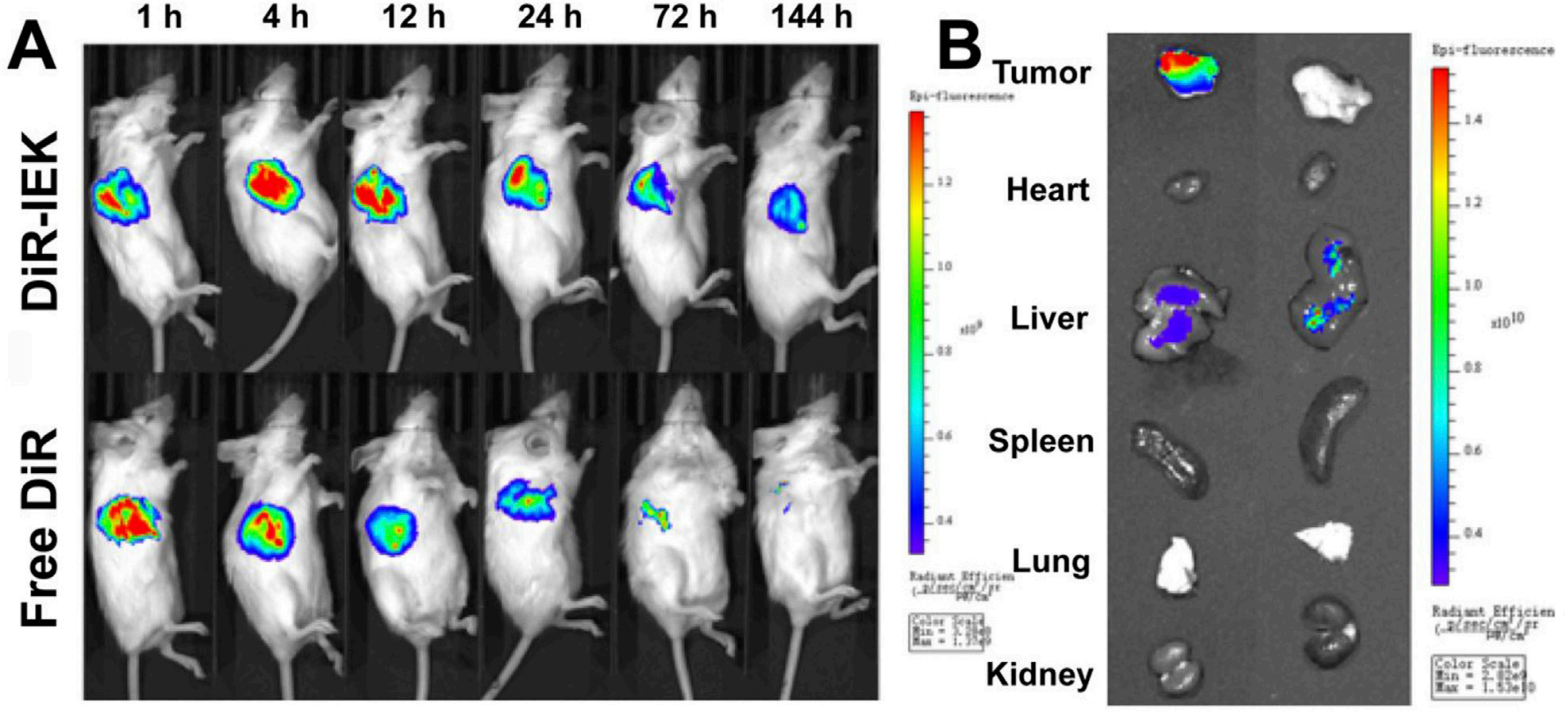
**(A)** *In vivo* fluorescence images of tumor-bearing mice after the administration of free DiR and DiR-IEK within the time range of 1–144 h. **(B)** *Ex vivo* fluorescence imaging of the tumor, heart, liver, spleen, lung, and kidney dissected from mice treated with free DiR and DiR-IEK for 144 h.

An animal fluorescence imaging system was used to visualize the biodistribution of the target drug released by the hydrogel *in vivo*. The free drug group showed the strongest DiR fluorescence signal within 1–4 h after free DiR injection. Unfortunately, fluorescence intensity declined rapidly over time and the fluorescence signal became difficult to detect at 72 h and 144 h. On the contrary, the DiR-IEK hydrogel group showed a strong fluorescence signal for a long period. The signal can still be clearly detected even at 144 h (Figure 5A). Compared with the free DiR group, the fluorescence distribution of main organs in the DiR-IEK group showed DiR concentrated in the tumor site, further proving that the IEK hydrogel can induce sustained release of the encapsulated drug and protect it from being degraded (Figure 5B).

### 3.8 *In vivo* anti-tumor efficacy

**FIGURE 6:**
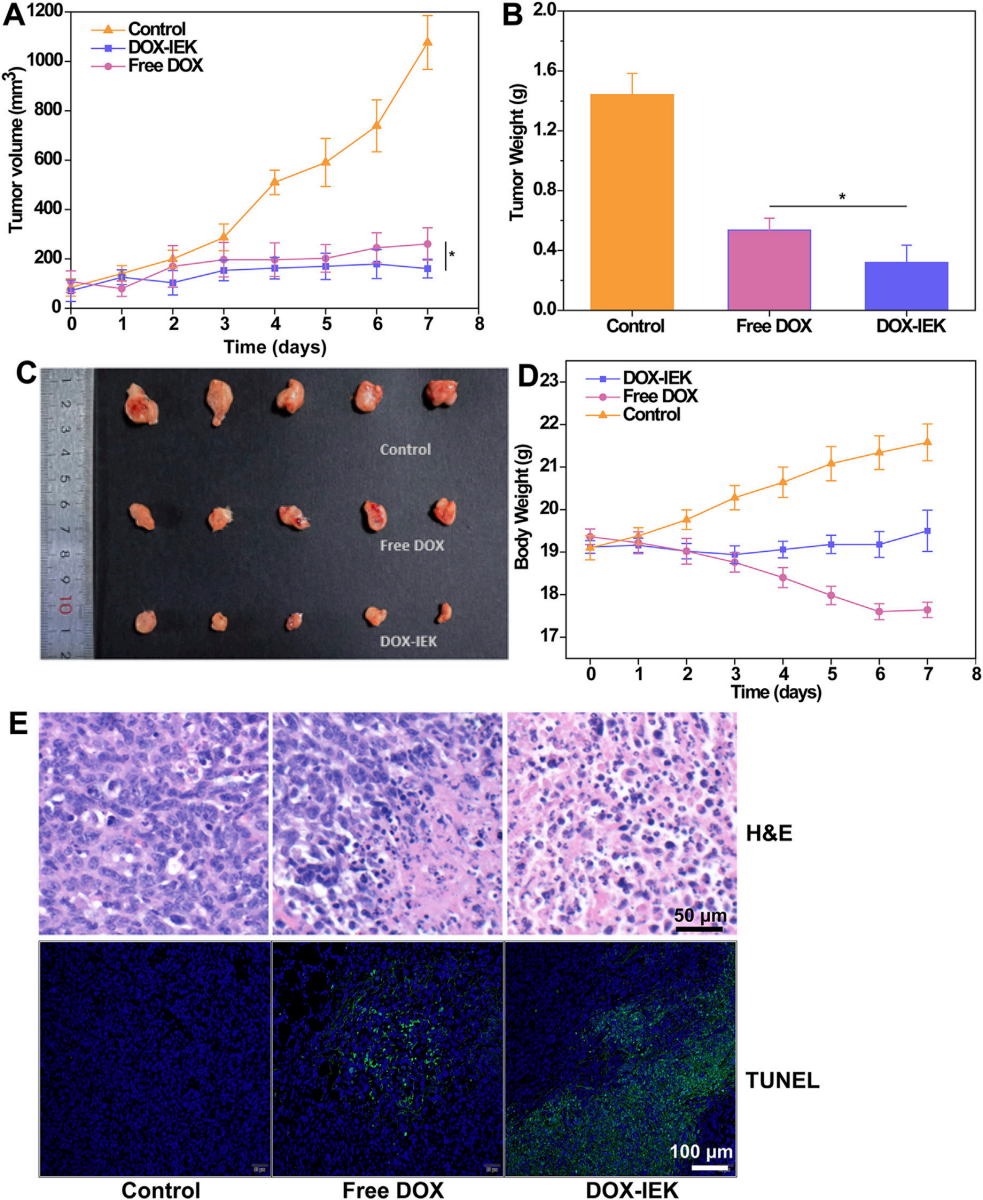
**(A)** Tumor volume curve of tumor-bearing mice during the treatment period. **(B)** Average tumor weight dissected after treatment for 7 days. **(C)** Image of tumor tissues dissected after treatment. **(D)** Body weight of mice within the treatment period. **(E)** H&E staining and **(F)** TUNEL assay images of tumor tissues dissected after treatment for 7 days. *, 0.01 < *p* < 0.05.

To further study the antitumor efficacy of the DOX-IEK hydrogel, NOS/SCID mice were inoculated with esophageal cancer cells *in situ* and treated with saline (control group), free DOX, and DOX-IEK. As shown in Figure 5A, compared with the blank control group, both free DOX and DOX-IEK groups were able to effectively inhibit the growth of tumor volume. At the end of the treatment cycle, the tumor volume of control group mice sharply increased to 1,000 mm^3^. It is worth noting that treatment efficacy of the free DOX group was better during the first 36 h (Figures 6A, C). However, the DOX-IEK group showed better efficacy after 36 h. This intriguing phenomenon may be due to the direct action of free drugs on tumor tissue, which quickly reduces tumor proliferation. However, due to the limited half-life, free DOX could not have a long-term effect. The IEK hydrogel served as a means of sustained and controlled release. In the acidic tumor microenvironment, the hydrogel gradually disintegrated, allowing for the stable release of DOX encapsulated within its network, achieving long-term anti-tumor efficacy. The average tumor mass of the control, free DOX, and DOX-IEK groups at the end of treatment was 1.44 g, 0.54 g, and 0.32 g, respectively, with a significant difference between the free DOX and DOX-IEK groups (Figure 6B), which was consistent with the statistics of tumor volume. The average body weight (Figure 6D) of the control group mice steadily increased from 19.1 g per day to 21.58 g per day. The value of DOX-IEK group mice did not show significant changes during the first 4 days but increased from the 5th day. Intriguingly, the weight of mice treated with free DOX gradually decreased from 19.36 g to 17.64 g, displaying a significant difference compared to the DOX-IEK group. DOX can play an anti-tumor role while producing certain toxic and side effects, thus reducing the quality of life of the organism. Therefore, the toxic reaction caused by DOX in mice free of drugs causes weight loss, while the IEK hydrogel can significantly reduce the adverse effects of DOX and improve the quality of life. In addition, to evaluate the therapeutic effect of the DOX-IEK hydrogel, we further carried out H&E and TUNEL staining. Compared with the control group, H&E staining of tumor tissues after the administration of DOX-IEK showed an enhanced degree of tissue necrosis (Figure 6E). TUNEL staining (Figure 6F) showed that the DOX-IEK group presented the largest percentage of apoptotic cells, which was consistent with the results of H&E staining (Figure 6E).

### 3.9 *In vivo* biocompatibility

H&E staining of the main organs dissected from healthy mice injected with saline (PBS group) and IEK hydrogel (IEK group) for 7 days showed no obvious pathological changes (inflammation or tissue necrosis), and routine blood tests showed no significant difference between the two groups, indicating that the IEK hydrogel had supreme biocompatibility *in vivo* (Supplementary Figures S6, S7).

## 4 Conclusion

In this research, we successfully developed a pH-responsive self-assembled peptide hydrogel loaded with anti-tumor drug doxorubicin. Studies showed that the drug delivery system had excellent self-assembly ability, high drug encapsulation rate, pH-responsive drug release behavior, and injectable/self-healing properties. After being injected into the acidic tumor microenvironment, the protonation degree of lysine directly lead to enhanced electrostatic repulsion and subsequent hydrogel dissociation. Released DOX can accumulate in tumor tissue and achieve anti-tumor efficacy. More importantly, the hydrogel can act as a drug reservoir for sustained drug release, improving drug-targeting ability and prolonging the duration of drug administration to compensate for the short half-life of DOX while reducing systematic toxicity. This method of drug delivery can be used not only for oncology treatment but also expanded to other diseases suitable for *in situ* drug delivery, such as rheumatoid arthritis. However, this pH-sensitive hydrogel drug delivery system still has certain limitations, such as the high cost of peptide synthesis, which restricts its clinical application. In addition, the nature of different anti-tumor drugs varies, making it difficult to extend this delivery method to all drugs. Nevertheless, it still remains a promising treatment option.

## Data Availability

The original contributions presented in the study are included in the article/Supplementary Material; further inquiries can be directed to the corresponding authors.
