# Supplementary material for: pH-sensitive supramolecular self-assembled peptide hydrogel for the treatment of esophageal cancer
Source: Front Pharmacol. 2024 Oct 24;15:1453422. doi: 10.3389/fphar.2024.1453422 (PMC11540713; doi:10.3389/fphar.2024.1453422)
Supplement: Supplementary file 1 [file DataSheet1.docx]

**pH-sensitive Supramolecular Self-assembled Peptide Hydrogel for the Treatment of Esophageal Cancer**

Gao-bing Ye^1,a^, Shiyao Luo^2,a^, Hajra Zafar^3^, Hong-lei Ge^1^ ,Binbin Liu^2^, Nan Wang^2^, Yu Jin^2^, Miao Wang^2^, Xu Chen^1,^* and Xiao-ming Ye^1,^*

a: These authors contributed equally to this work

*: Co-corresponding authors

^1^ Afﬁliated Yueqing Hospital, Wenzhou Medical University, Wenzhou, Zhejiang, 325600, China

^2^State Key Laboratory of Natural Medicines, School of Pharmacy, China Pharmaceutical University, Nanjing, Jiangsu, 210009, China.

^3^ School of Pharmacy, Shanghai Jiao Tong University, 800 Dongchuan Road, 200240, Shanghai, China

***Corresponding authors**

**Xu Chen**, E-mail address:[709979053@qq.com](mailto:709979053@qq.com)

**Xiao-ming Ye,**  E-mail address:[1359582158@qq.com](mailto:1359582158@qq.com)





Fig. S1 Brief process of solid phase peptide synthesis.


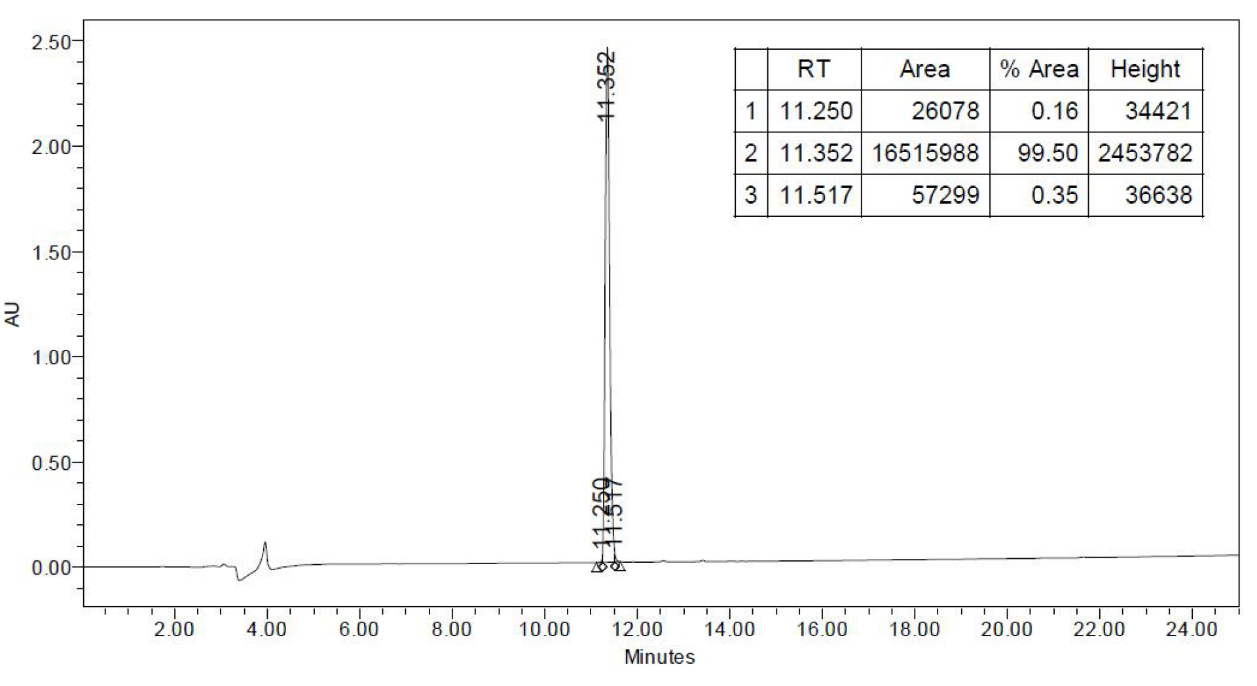


Fig. S2 HPLC spectrum of IEK peptide.


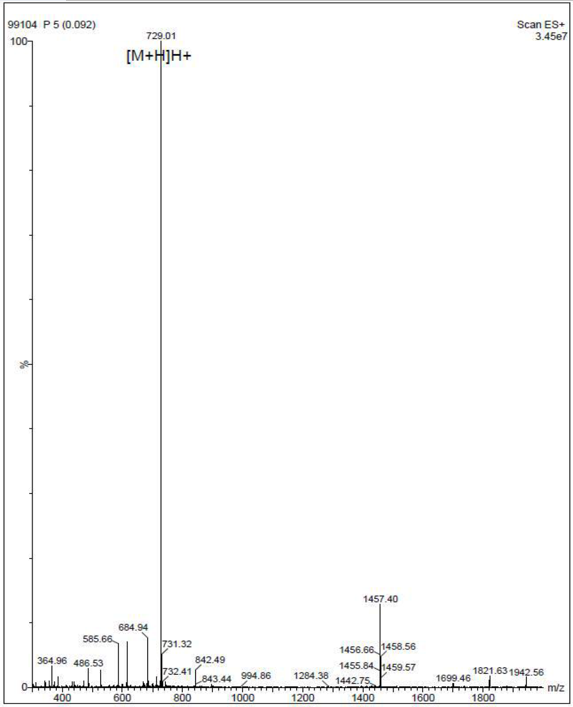


Fig. S3 LC-MS spectrum of IEK peptide.

**IEK**


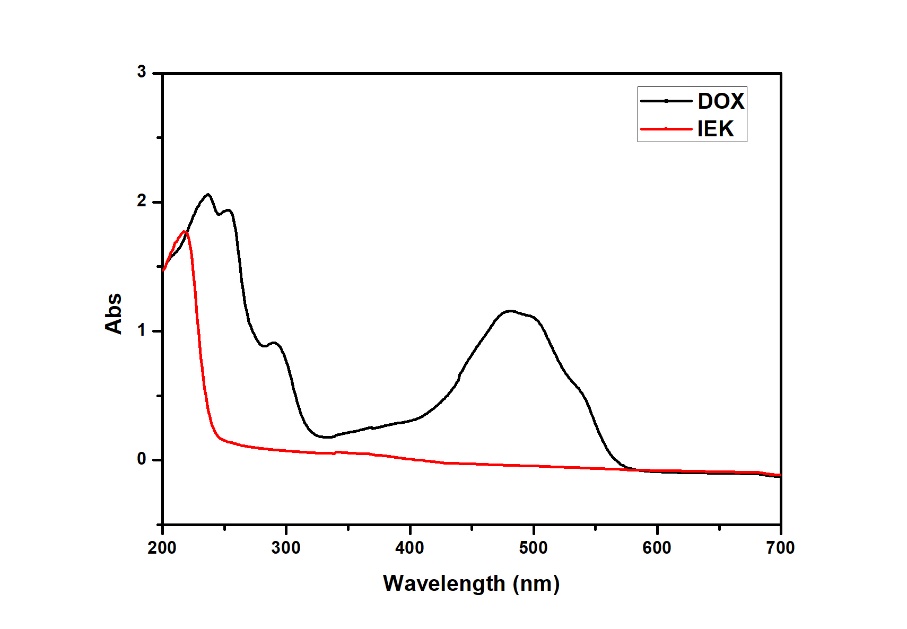


Fig. S4 UV/vis spectrum of DOX and IEK peptide.


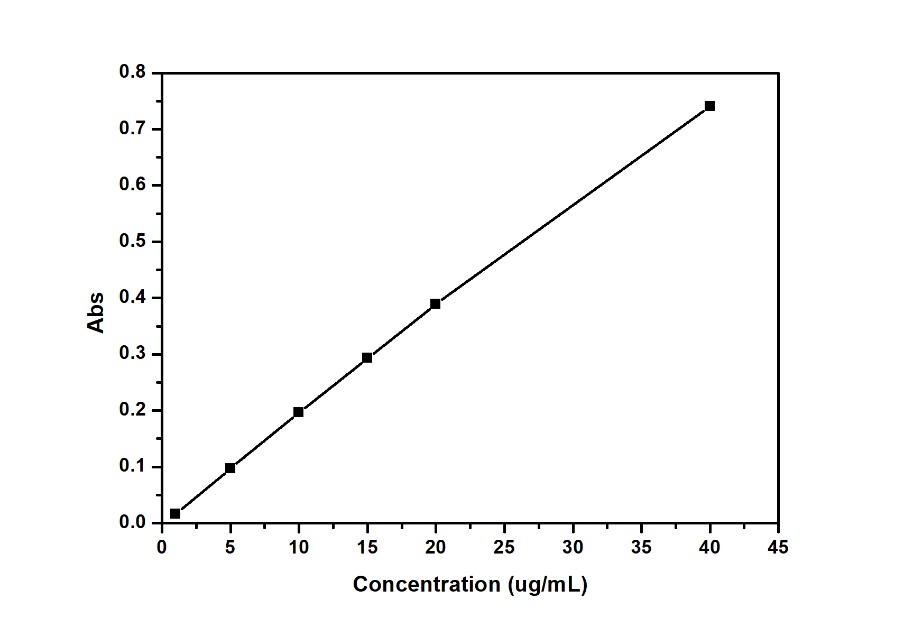


Fig. S5 UV/vis standard curve of DOX.


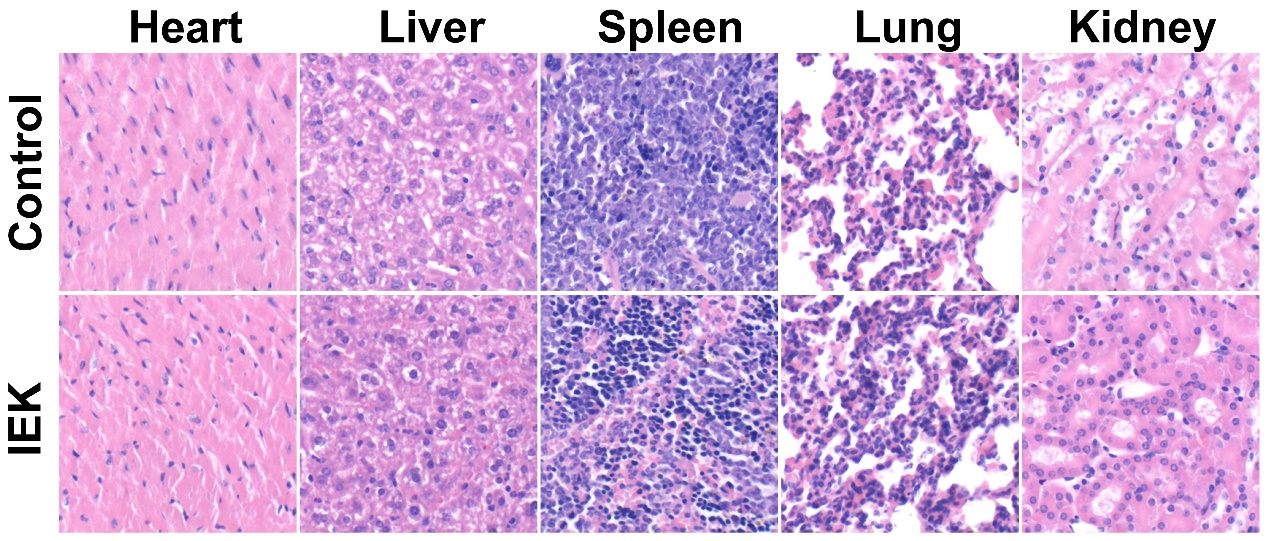


Fig. S6 H&E staining of main organs dissected from healthy mice injected with saline (Control group) and blank IEK peptide.


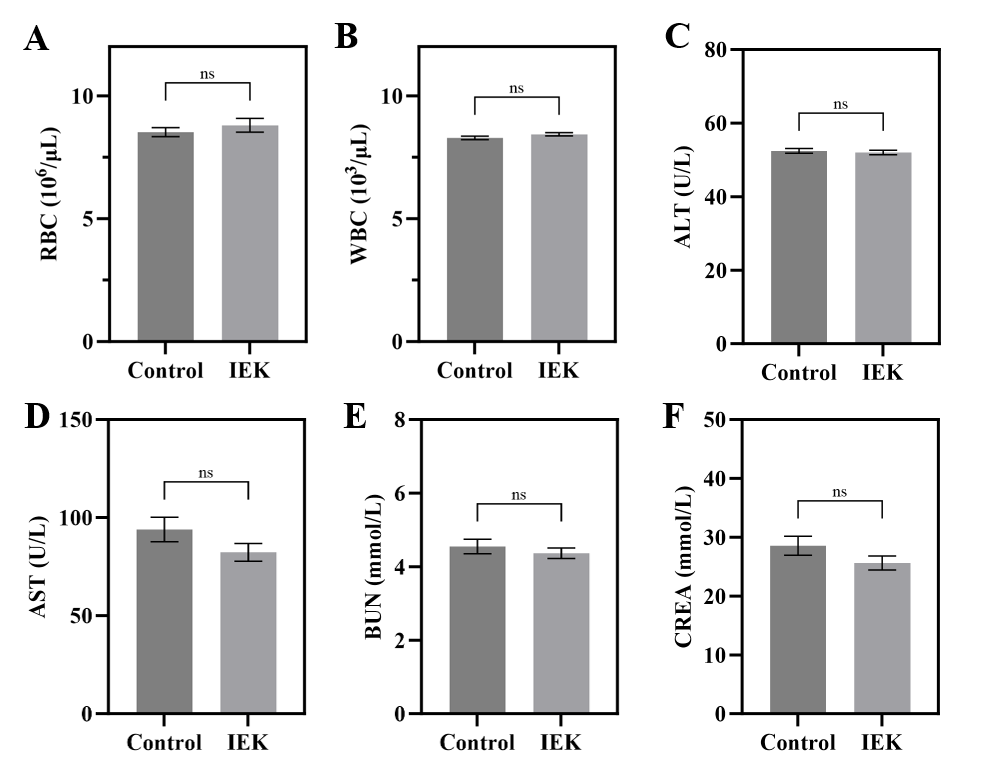


Fig. S7 Routine blood tests of mice. (A)RBC; (B)WBC; (C)ALT; (D)AST; (E)BUN; (F)CREA. ns, no significance, p ＞ 0.05.
